# Supplementary material for: Non-centering for discrete-valued state transition models: an application to ESBL-producing E. coli transmission in Malawi
Source: Stat Comput. 2026 Aug 20;36(5):208. doi: 10.1007/s11222-026-10956-z (PMC13493405; doi:10.1007/s11222-026-10956-z)
Supplement: Supplementary file 1 [file 11222_2026_10956_MOESM1_ESM.pdf]

Non-centering for discrete-valued state transition models: an application to ESBL-producing *E. coli* transmission in Malawi – Supplementary Materials

S1 Rippler Algorithm

S1.1 AntiDOTE Data Analysis

In the AntiDOTE study, the 478 individuals who were sampled are split between 110 houses, with the distribution of house sizes shown in Figure S1 (left). We see from the histogram in Figure S1 (right) that the ages of individuals (at the time they were first tested) were right-skewed, with a a majority of individuals under the age of 18. The test results for each individual over the 62 weeks are shown in Figure S2, with 5.60% of entries containing a sample. We can clearly see when each individual was initially sampled, and then follow-up tests one, three, and six months later.

The convergence of the algorithm is checked using the Gelman-Rubin statistic. We run three MCMC chains, as in Section 5, each with different starting parameter values $\boldsymbol{\theta}^{(0)}$: $(0.5, 0.5, 0, 0)$, $(0.02, 0.75, -0.02, -0.5)$, and $(0.2, 4, 0.02, 0.5)$. The starting latent variables $\boldsymbol{X}^{(0)}$ for each chain are generated from the transmission model using the corresponding $\boldsymbol{\theta}^{(0)}$. The first 20,000 values are discarded as burn-in. The Gelman-Rubin statistics for comparing the posterior samples of $\boldsymbol{\theta}$ across the three chains are $\hat{R} = 1.009$ for β_G, $\hat{R} = 1.008$ for β_H, $\hat{R} = 1.011$ for δ_A, and $\hat{R} = 1.082$ for δ_S. For each parameter we have $\hat{R} < 1.1$, indicating the chains have converged to the posterior distributions (Gelman et al., 2004).

The parameter trace plots when using the Rippler algorithm for inference on the AntiDOTE test data are shown in Figure S3. For full details of the AntiDOTE data analysis see the main text.

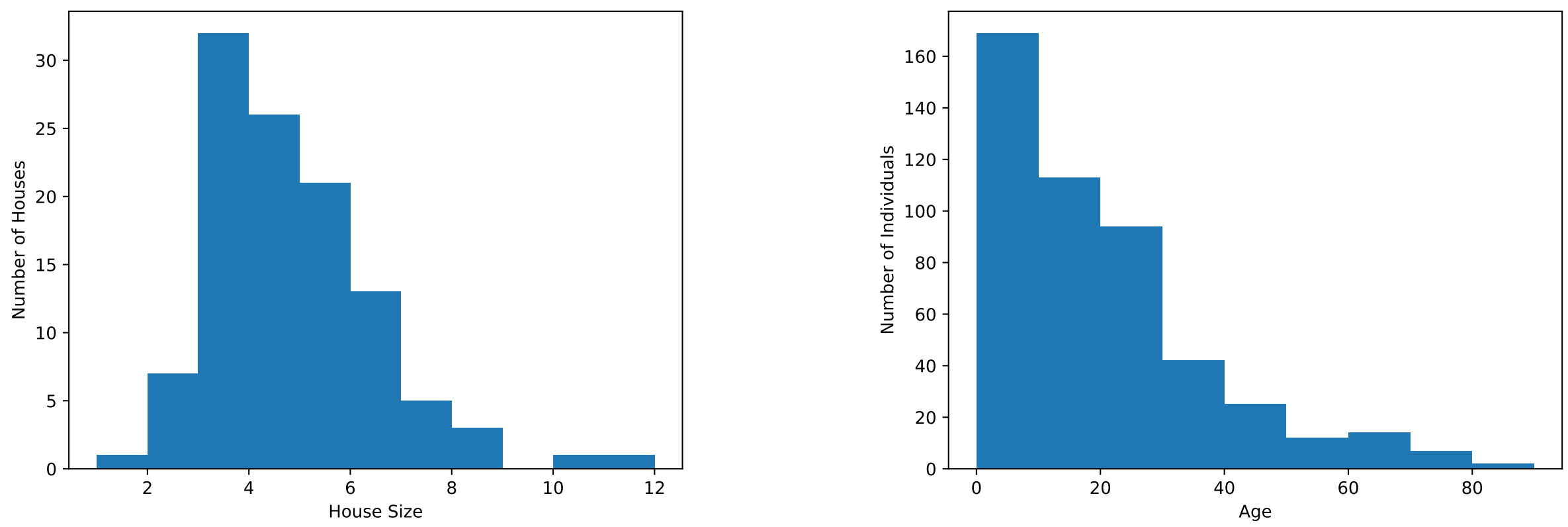

Figure S1: Household size (left) and age data (right) from the AntiDOTE study.

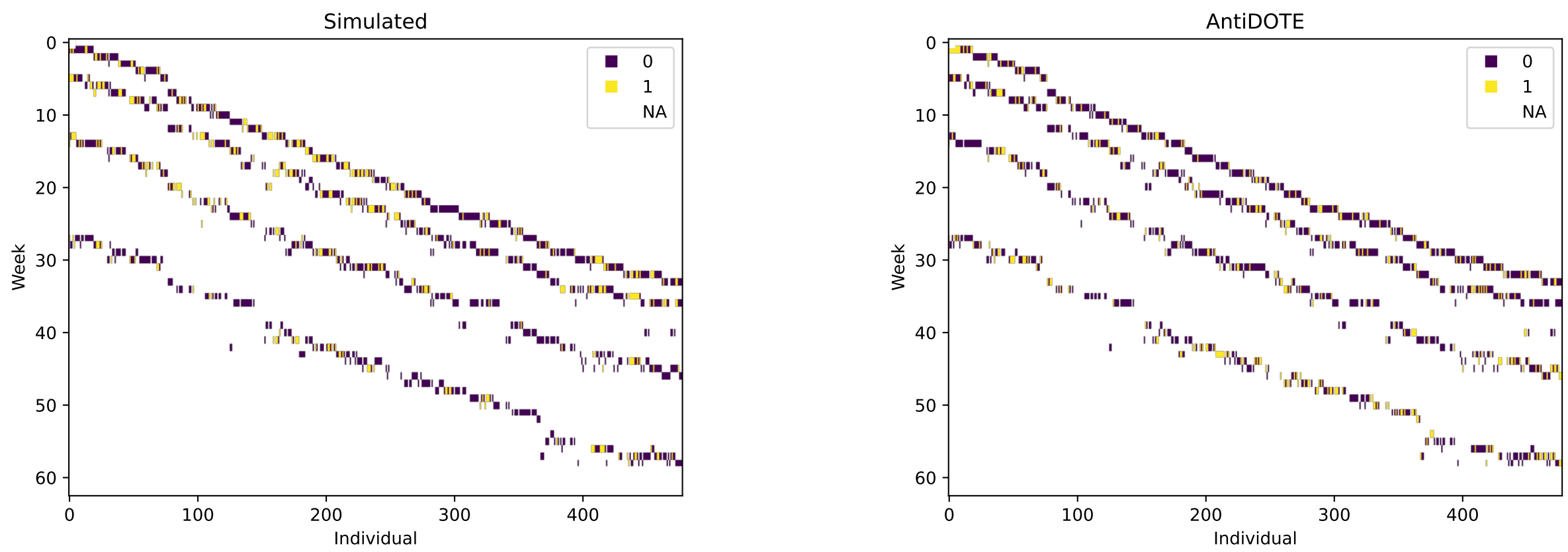

Figure S2: Test results for 3GC-R *E. coli* using simulated test data (left) and test data from the AntiDOTE study (right). Yellow and purple squares represent positive and negative test results respectively.

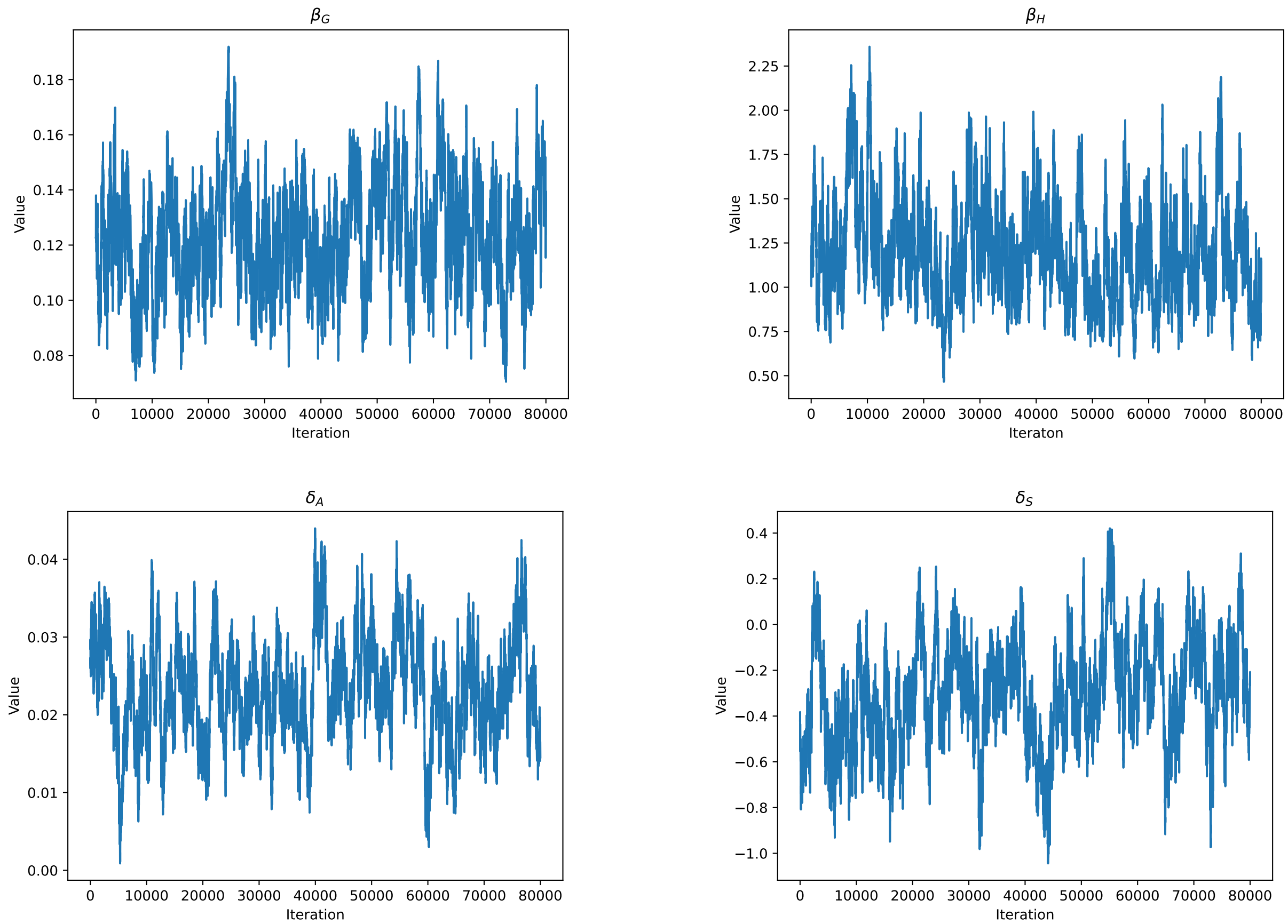

Figure S3: Trace plots of the posterior samples generated for the parameters $\boldsymbol{\theta} = (\beta_G, \beta_H, \delta_A, \delta_S)$ for 3GC-R *E. coli* transmission using test data from the AntiDOTE study using the Rippler algorithm.

S1.2 Simulation Study

The mean value of each latent variable when using the Rippler algorithm for inference on the simulated test data is shown in Figure S4, along with the underlying "true" simulation from the transmission model. We see that the Rippler algorithm is broadly able to identify the latent variables, with greater mean values in regions with more colonised individuals/time-points. In particular, the few households that are often colonised during the dry season are identified. We also see the four times each individual is tested as spots where the mean latent variables are close to 0 or 1; the shape of the bright spots closely matches Figure S2. This pattern does not appear on the underlying colonisation data because only the transmission model was used to simulate this data, not the observation model.

The convergence of the algorithm is checked using the Gelman-Rubin statistic. We run three MCMC chains, as in Section 4.2, each with different starting parameter values $\boldsymbol{\theta}^{(0)}$: $(0.5, 0.5, 0, 0)$, $(0.02, 0.75, -0.02, -0.5)$, and $(0.2, 4, 0.02, 0.5)$. The starting latent variables $\boldsymbol{X}^{(0)}$ for each chain are generated from the transmission model using the corresponding $\boldsymbol{\theta}^{(0)}$. The first 10,000 values are discarded as burn-in. The Gelman-Rubin statistics for comparing the posterior samples of $\boldsymbol{\theta}$ across the three chains are $\hat{R} = 1.003$ for β_G, $\hat{R} = 1.008$ for β_H, $\hat{R} = 1.003$ for δ_A, and $\hat{R} = 1.067$ for δ_S. For each parameter we have $\hat{R} < 1.1$, indicating the chains have converged to the posterior

distributions (Gelman et al., 2004).

An additional check on the consistency of the algorithm is testing the Rippler algorithm on 100 simulations from the UC model. All 100 simulations use the same parameters $\boldsymbol{\theta} = (0.1, 1.5, 0, 0)$, but different random number seeds for generating $\boldsymbol{X}$ and $\boldsymbol{Y}$. The number of colonised individuals over time for the 100 different latent variables $\boldsymbol{X}$ is shown in Figure S7. Posterior samples were generated by running the Rippler algorithm for $K = 11{,}000$ iterations on each of the 100 simulations, with the first 1000 values discarded as burn-in. The 95% credible intervals and medians of the posterior samples are shown in Figure S8. Out of the 100 runs, the true value of the parameter was inside the central 95% credible interval 95 times for β_G, 93 times for β_H, 89 times for δ_A, and 99 times for δ_S.

Figure S5 shows the parameter trace plots when using the Rippler algorithm for inference on the simulated test data. For full details of the simulation study using the Rippler algorithm see the main text. Figure S6 shows the posterior mean of $x_{t,j}$ for for each pair (t, j), comparing the results when using the Rippler or iFFBS algorithms for inference. We see that the posterior mean values are approximately the same for both methods, suggesting that both methods are sampling from the same posterior distribution (with small differences that can be attributed to Monte Carlo error).

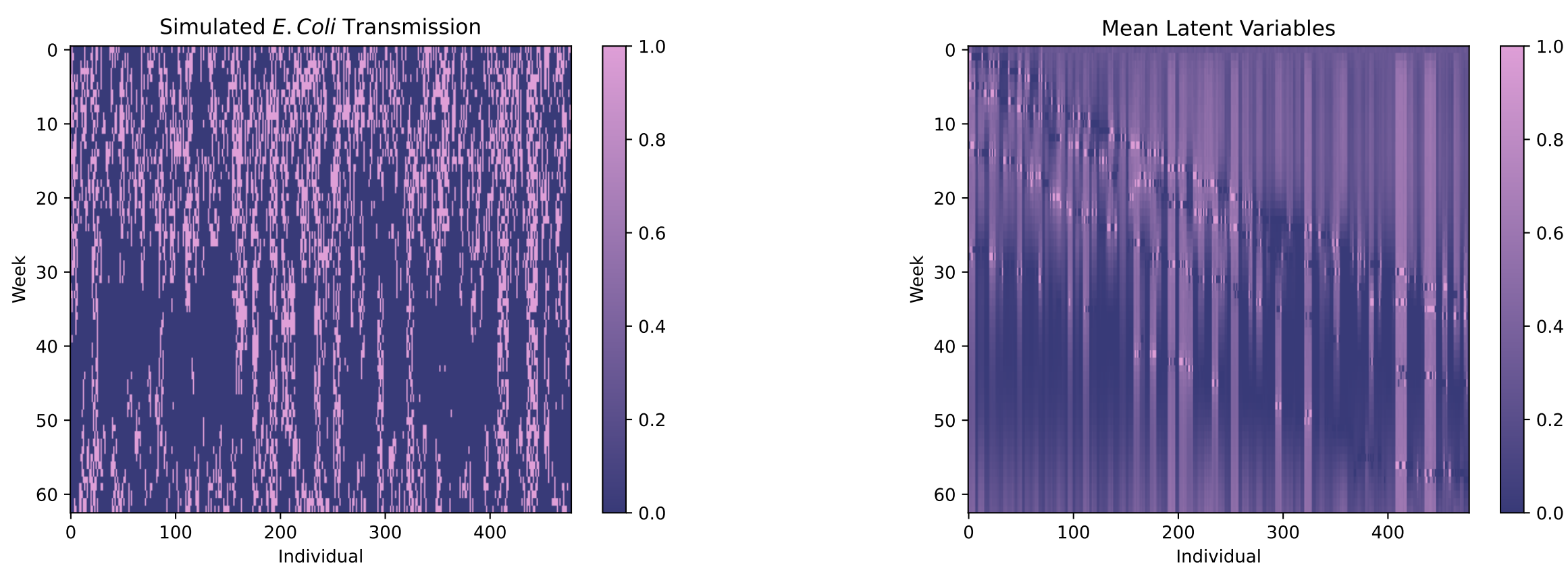

Figure S4: The underlying "true" simulation from the transmission model (left) and the mean value of each latent variable (right) for simulated 3GC-R *E. coli* test data using the Rippler algorithm.

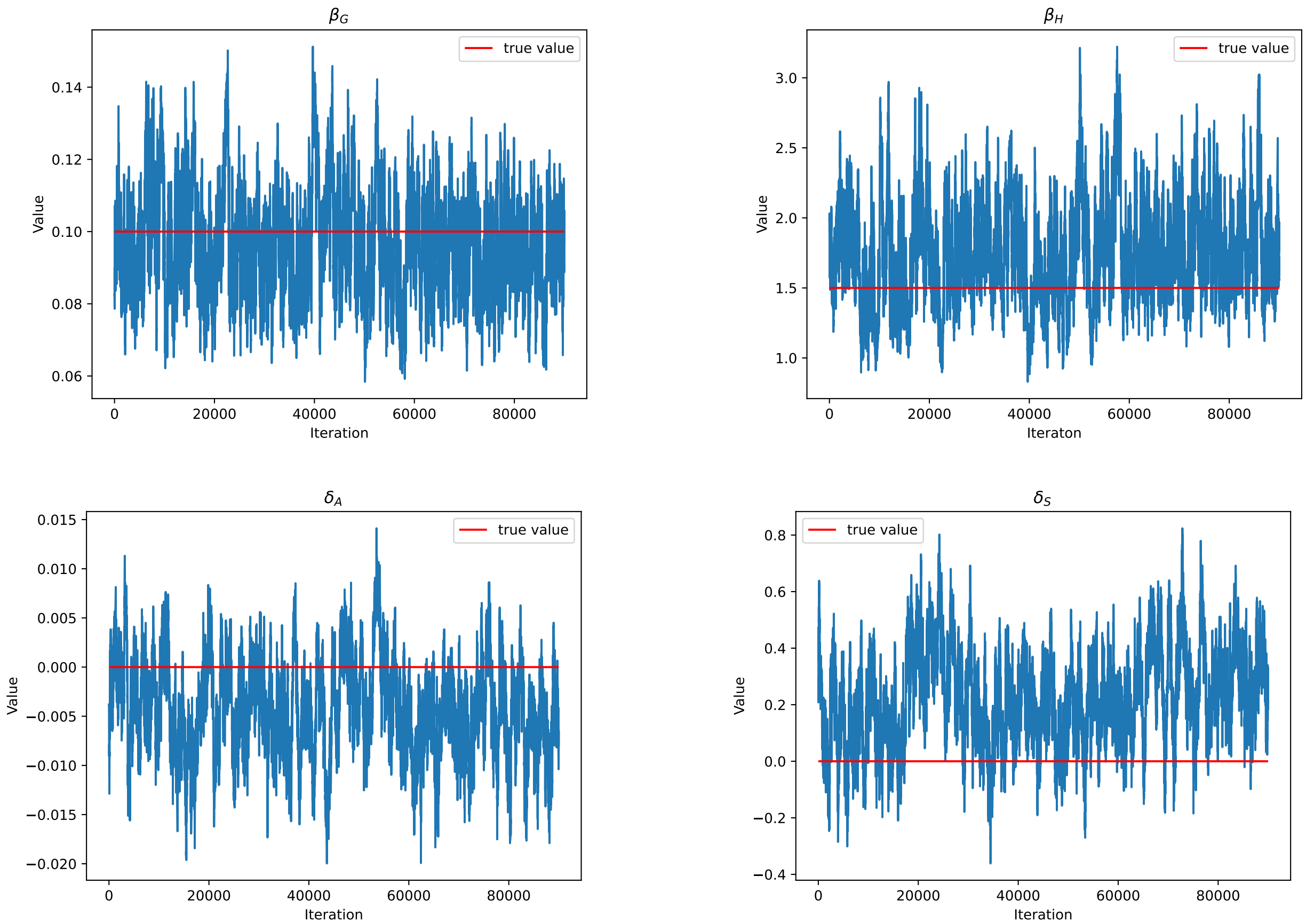

Figure S5: Trace plots of the posterior samples generated for the parameters $\boldsymbol{\theta} = (\beta_G, \beta_H, \delta_A, \delta_S)$ for simulated 3GC-R *E. coli* test data using the Rippler algorithm. The true value is shown by the red line.

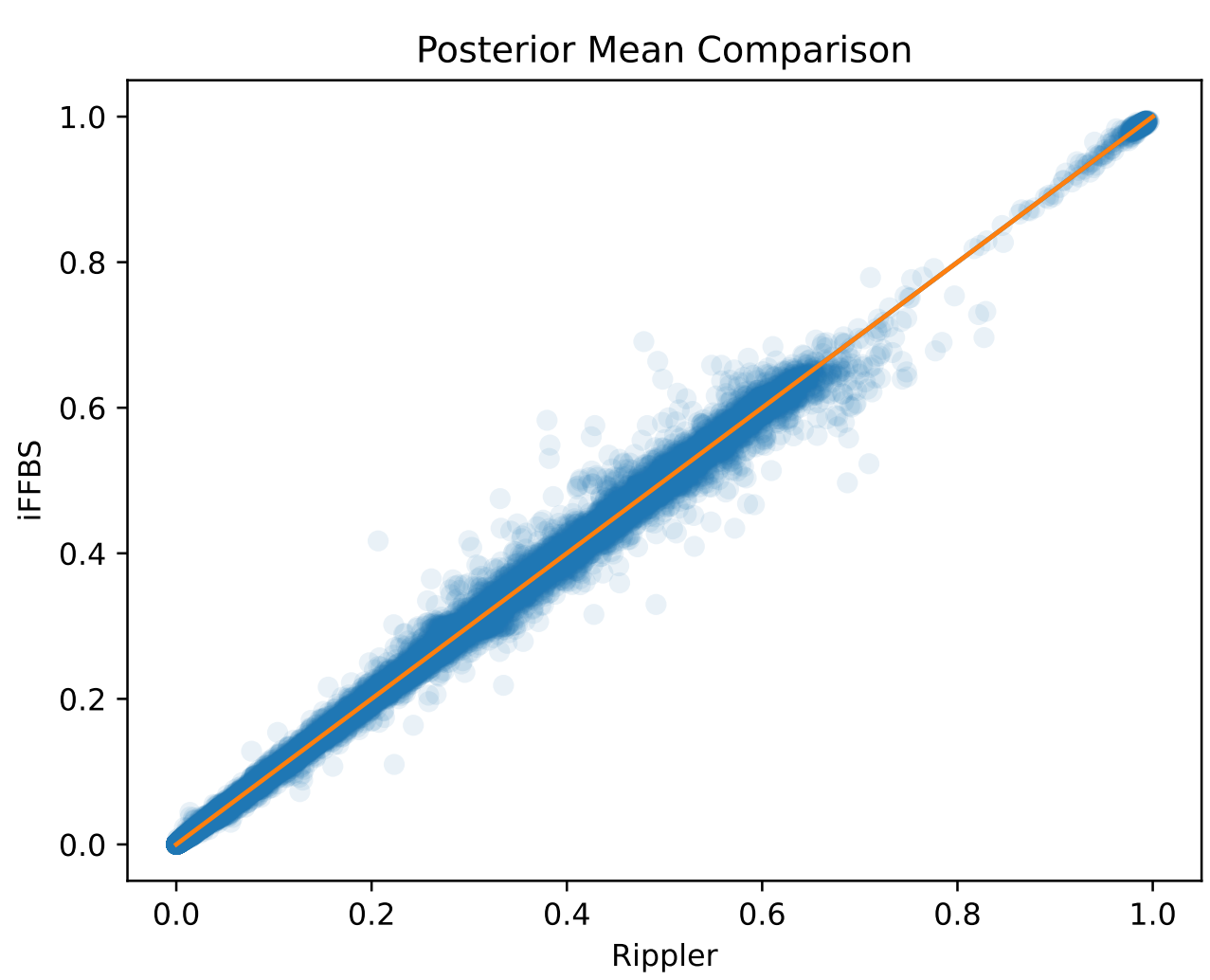

Figure S6: Comparison of the posterior mean of $x_{t,j}$ for each pair (t, j) between the Rippler and iFFBS inference methods.

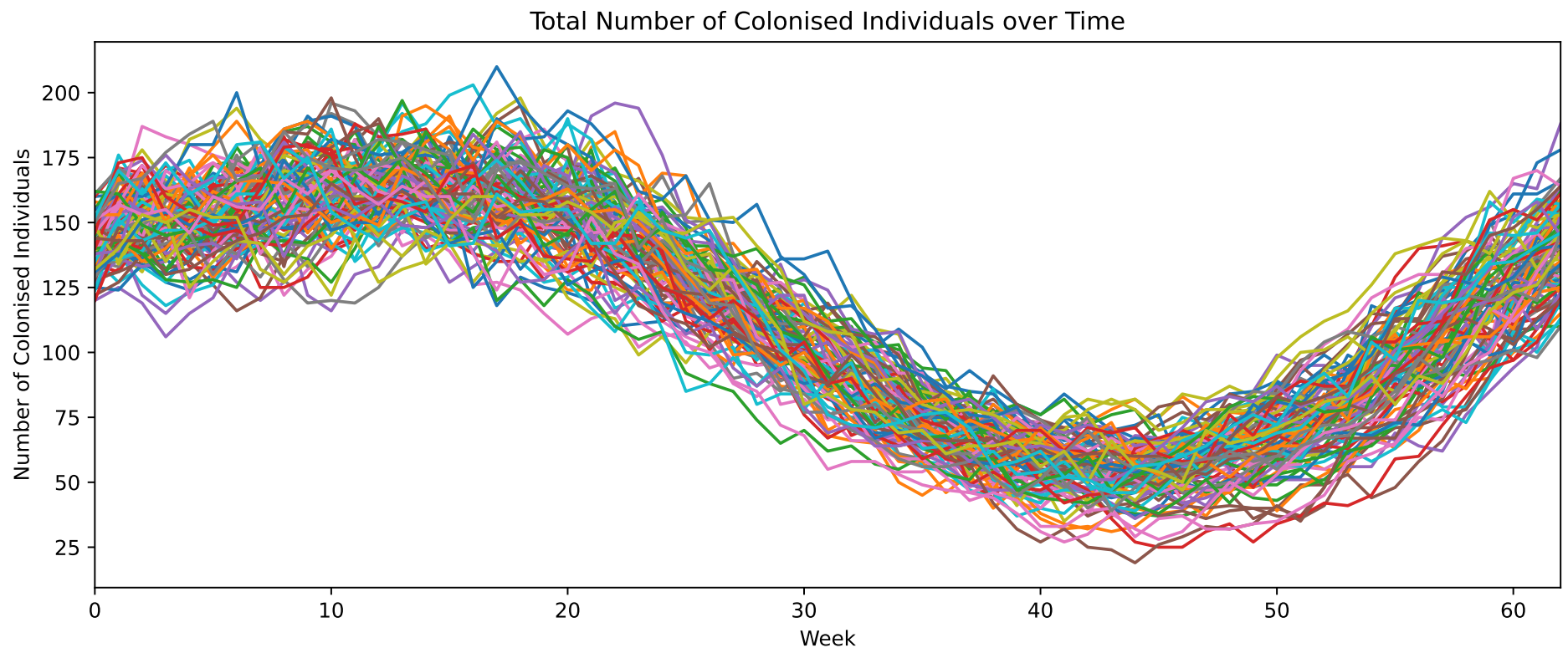

Figure S7: The total number of colonised individuals over time in 100 simulations from the UC model, all with $\boldsymbol{\theta} = (0.1, 1.5, 0, 0)$.

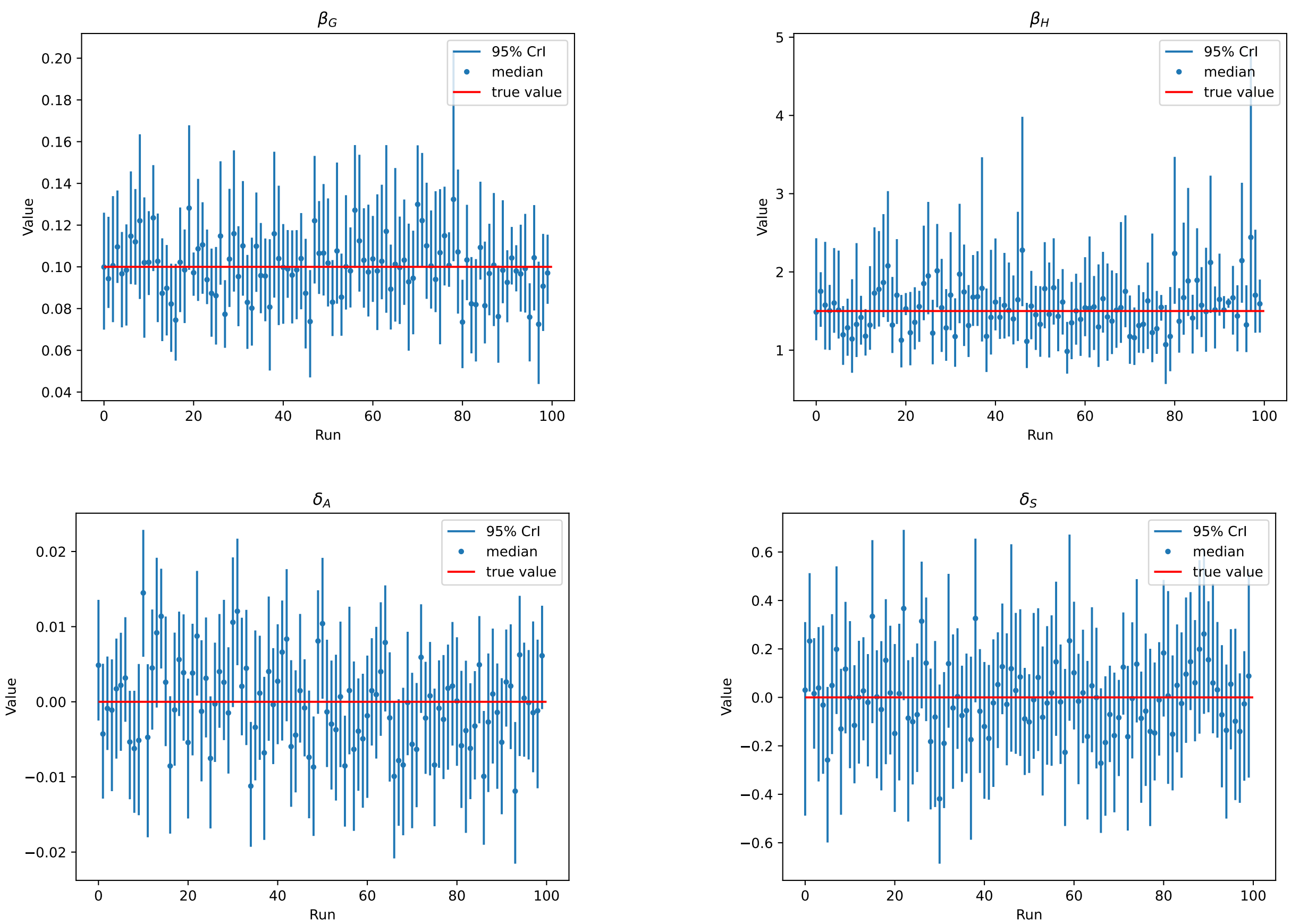

Figure S8: 95% credible intervals and medians of the posterior samples for the parameters $\boldsymbol{\theta} = (\beta_G, \beta_H, \delta_A, \delta_S)$ for 100 simulated 3GC-R *E. coli* test data. The true value is shown by the red line.

S1.3 Comparison with existing inference methods

In Section 4.3 we compared the mean squared jump distance (MSJD) of the three methods tested for inference on the simulated *E. coli* test data: the Rippler algorithm, reversible-jump MCMC, and iFFBS. Table S1 expands this comparison to include the run time in hours and MSJD per hour taken of the three methods. We see that the relative efficiency of the three methods is maintained when comparing MSJD per hour taken: the MSJD per hour is 6.65 for the reversible-jump algorithm, 17.7 for the Rippler algorithm, and 131 for the iFFBS algorithm.

Table S1: Comparison of the MJSD, run time in hours, and MSJD per hour between the Rippler, reversible-jump MCMC, and iFFBS methods.

Method	MSJD	Run time in hours	MSJD per hour
Reversible-jump	244	36.7	6.65
Rippler	1730	97.8	17.7
iFFBS	4350	33.2	131

S1.4 Rippler Tuning

We also tested how the acceptance rate and mixing of the Rippler algorithm changes as we vary the number of elements of the random number matrix $\boldsymbol{U}$ that are altered in the proposal step. Let the number of elements in the random number matrix to alter be K''. Then we follow Algorithm 4 as normal, but repeat the process in steps 7-9 K'' times. This means we must also change the proposal part of the $\alpha(\boldsymbol{X}, \boldsymbol{X}^*)$ calculation in step 11 to

$$q_2(\boldsymbol{U}^*|\boldsymbol{\theta}, \boldsymbol{U}, \boldsymbol{X}) = \frac{1}{\left(\sum_{t=0}^{T} \sum_{j=1}^{N} (1 + a_{t,j} - b_{t,j})\right)^{K''}}.$$

We see how this affects the MSJD in Figure S9 (left), with the MSJD maximised when 2 elements of $\boldsymbol{U}$ are changed in each step. In Figure S9 (right), we see that the acceptance rate decreases as the the number of altered elements of $\boldsymbol{U}$ increases; the greater difference between $\boldsymbol{U}$ and $\boldsymbol{U}^*$ leads to a greater difference between $\boldsymbol{X}$ and $\boldsymbol{X}^*$, and so it makes sense that this causes the acceptance rate to decrease. The size of changes to $\boldsymbol{X}$ that are likely to be accepted depends on the density of the observed data; we expect that with sparser observation data bigger moves to $\boldsymbol{X}$ would be more likely to be accepted, and so a greater number of altered elements of $\boldsymbol{U}$ would be optimal to maximise the MSJD.

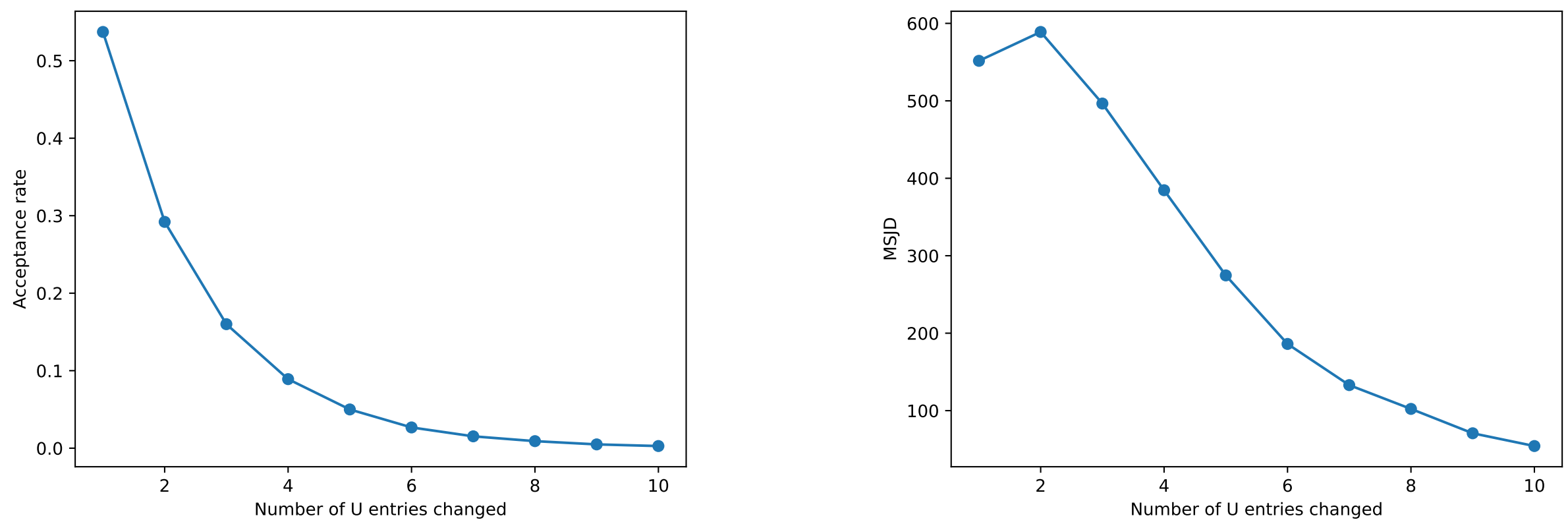

Figure S9: Changes to the acceptance rate (left) and MSJD (right) as the number of altered elements of the random number matrix in the Rippler proposal increases.

S2 Reversible-jump MCMC

S2.1 Inference Algorithm

We use the method from Spencer et al. (2015) to perform reversible-jump MCMC. As with the Rippler algorithm, we use a Metropolis-within-Gibbs approach to update our parameters and latent variables separately. We use the same method of updating the parameters $\boldsymbol{\theta}$ as in the Rippler algorithm (an adaptive random walk Metropolis algorithm). The initial conditions are updated separately from the rest of the latent variables, so we now derive both $\pi(\boldsymbol{x}_0|\boldsymbol{\theta}, \boldsymbol{X}_{1:T}, \boldsymbol{Y})$ and $\pi(\boldsymbol{X}_{1:T}|\boldsymbol{\theta}, \boldsymbol{x}_0, \boldsymbol{Y})$.

By Bayes' theorem we have

$$\pi(\boldsymbol{\theta}, \boldsymbol{X}|\boldsymbol{Y}) \propto \pi(\boldsymbol{Y}|\boldsymbol{X}, \boldsymbol{\theta})\pi(\boldsymbol{X}|\boldsymbol{\theta})\pi(\boldsymbol{\theta}).$$

We know that $\pi(\boldsymbol{X}|\boldsymbol{\theta}, \boldsymbol{Y}) \propto \pi(\boldsymbol{\theta}, \boldsymbol{X}|\boldsymbol{Y})$. Let $N_0(\boldsymbol{X}) = \sum_{j=1}^{N} x_{0,j}$. Then from the main text we have

$$\begin{aligned}\pi(\boldsymbol{X}|\boldsymbol{\theta}, \boldsymbol{Y}) \propto\ & s_e^{TP(\boldsymbol{X},\boldsymbol{Y})}(1-s_e)^{FN(\boldsymbol{X},\boldsymbol{Y})} s_p^{FP(\boldsymbol{X},\boldsymbol{Y})}(1-s_p)^{TN(\boldsymbol{X},\boldsymbol{Y})} \\ & \prod_{(t,j)\in UC(\boldsymbol{X})} (1-e^{-\lambda_j(t,\boldsymbol{x}_{t-1},\boldsymbol{\theta})}) \prod_{(t,j)\in UU(\boldsymbol{X})} e^{-\lambda_j(t,\boldsymbol{x}_{t-1},\boldsymbol{\theta})} \\ & (1-e^{-\gamma})^{|CU(\boldsymbol{X})|}\, e^{-\gamma|CC(\boldsymbol{X})|}\, p_0^{N_0(\boldsymbol{X})}\, (1-p_0)^{N-N_0(\boldsymbol{X})}.\end{aligned}$$

This means we have

$$\begin{aligned}\pi(\boldsymbol{x}_0|\boldsymbol{\theta}, \boldsymbol{X}_{1:T}, \boldsymbol{Y}) \propto & \prod_{(t,j)\in UC(\boldsymbol{X}_{0:1})} (1-e^{-\lambda_j(t,\boldsymbol{x}_{t-1},\boldsymbol{\theta})}) \prod_{(t,j)\in UU(\boldsymbol{X}_{0:1})} e^{-\lambda_j(t,\boldsymbol{x}_{t-1},\boldsymbol{\theta})} \\ & (1-e^{-\gamma})^{|CU(\boldsymbol{X}_{0:1})|}\, e^{-\gamma|CC(\boldsymbol{X}_{0:1})|}\, p_0^{N_0(\boldsymbol{X})}\, (1-p_0)^{N-N_0(\boldsymbol{X})},\end{aligned} \tag{S1}$$

and

$$\pi(\boldsymbol{X}_{1:T}|\boldsymbol{\theta},\boldsymbol{x}_0,\boldsymbol{Y}) \propto s_e^{TP(\boldsymbol{X},\boldsymbol{Y})}(1-s_e)^{FN(\boldsymbol{X},\boldsymbol{Y})} s_p^{FP(\boldsymbol{X},\boldsymbol{Y})}(1-s_p)^{TN(\boldsymbol{X},\boldsymbol{Y})} \prod_{(t,j)\in UC(\boldsymbol{X})}(1-e^{-\lambda_j(t,\boldsymbol{x}_{t-1},\boldsymbol{\theta})}) \prod_{(t,j)\in UU(\boldsymbol{X})} e^{-\lambda_j(t,\boldsymbol{x}_{t-1},\boldsymbol{\theta})} (1-e^{-\gamma})^{|CU(\boldsymbol{X})|}\ e^{-\gamma|CC(\boldsymbol{X})|}. \tag{S2}$$

We propose a new value $\boldsymbol{x}_0^*$ by randomly choosing one individual j and setting $x_{0,j}^* = 1 - x_{0,j}$. For all other individuals k, we keep $x_{0,k}^* = x_{0,k}$. The probability of any one move is $1/N$, so the proposal ratio is

$$\frac{q(\boldsymbol{x}_0|\boldsymbol{x}_0^*)}{q(\boldsymbol{x}_0^*|\boldsymbol{x}_0)} = \frac{1/N}{1/N} = 1.$$

This means the acceptance probability is

$$\alpha(\boldsymbol{x}_0,\boldsymbol{x}_0^*) = \min\left\{1, \frac{\pi(\boldsymbol{x}_0^*|\boldsymbol{\theta},\boldsymbol{X}_{1:T},\boldsymbol{Y})}{\pi(\boldsymbol{x}_0|\boldsymbol{\theta},\boldsymbol{X}_{1:T},\boldsymbol{Y})}\right\},$$

which we calculate using Equation S1.

We propose a new value $\boldsymbol{X}_{1:T}^*$ by proposing alterations to the colonisation and recovery events in the epidemic. At each step, we make one of three possible changes: move a colonisation/recovery time, add a colonisation/recovery time, or remove a colonisation/recovery time.

Which of the three possible changes to propose is chosen randomly (probability 1/3), and is proposed for a random individual j (probability $1/N$). For the chosen individual j, let $(t_1,\dots,t_E)$ be the times when colonisation or recovery occur (where E is the total number of colonisation and recovery events). We limit the sizes of the changes to the duration of colonisation/recovery periods to some value m, chosen to maximise the efficiency of the algorithm.

When moving an event, we take the following steps:

1. Randomly choose e from $\{1,\dots,E\}$. We will be proposing a new time for event e.
2. If $e=1$, then let $t_0 = 0$. If $e=E$, then let $t_{E+1} = T+1$.
3. Let $F = \{t_{e-1}+1,\dots,t_{e+1}-1\}$, all possible times between the previous and next events.
4. Let $G = \{t_e - m,\dots,t_e+m\}$, all possible times within the update limit.
5. Then let $H = (F\cap G)\backslash\{t_e\}$, the set of possible times to move event e to.
6. Randomly choose t_e^* from H (this is the new time for event e).
7. If $t_e^* > t_e$, then let $x_{t,j}^* = 1 - x_{t,j}$ for $t\in\{t_e+1,\dots,t_e^*\}$. If $t_e^* < t_e$, then let $x_{t,j}^* = 1-x_{t,j}$ for $t\in\{t_e^*,\dots,t_e-1\}$. All other entries in $\boldsymbol{X}_{1:T}^*$ are equal to $\boldsymbol{X}_{1:T}$.

This means our proposed $\boldsymbol{X}_{1:T}^*$ occurs with probability $1/(3NE|H|)$.

When adding an event, we take the following steps:

1. Let $A = \{t : x_{t-1,j} = x_{t,j} = x_{t+1,j}\}$, the set of acceptable new event times.
2. Randomly choose t_a from A. This is the time where a new event will be added.

3. Let $e' = \min\{e : t_e > t_a\}$, the next event after time t_a.
4. Let $B = \{t_a + 1, \ldots, \min\{t_{e'} - 1, t_a + m\}\}$, the set of acceptable event times to reverse the newly added event.
5. Randomly choose t_b from B. This is the time when another new event will be added.
6. Let $x^*_{t,j} = 1 - x_{t,j}$ for $t \in \{t_a, \ldots, t_b - 1\}$. All other entries in $\boldsymbol{X}^*_{1:T}$ are equal to $\boldsymbol{X}_{1:T}$.

This means our proposed $\boldsymbol{X}^*_{1:T}$ occurs with probability $1/(3N|A||B|)$.

When removing an event, we take the following steps:

1. Let $R = \{e : t_e + m \geq t_{e+1}\}$, the set of events we can remove.
2. Randomly choose r from R. This is the event we will remove, along with event $r + 1$.
3. Let $x^*_{t,j} = 1 - x_{t,j}$ for $t \in \{t_r, \ldots, t_{r+1} - 1\}$. All other entries in $\boldsymbol{X}^*_{1:T}$ are equal to $\boldsymbol{X}_{1:T}$.

This means our proposed $\boldsymbol{X}^*_{1:T}$ occurs with probability $1/(3N|R|)$.

The reverse step to a move event is another move event, so the proposal ratio is

$$Q = \frac{q(\boldsymbol{X}_{1:T}|\boldsymbol{X}^*_{1:T})}{q(\boldsymbol{X}^*_{1:T}|\boldsymbol{X}_{1:T})} = \frac{1/(3NE|H^*|)}{1/(3NE|H|)} = \frac{|H|}{|H^*|}.$$

The reverse step to an add event is a remove event, so the proposal ratio is

$$Q = \frac{q(\boldsymbol{X}_{1:T}|\boldsymbol{X}^*_{1:T})}{q(\boldsymbol{X}^*_{1:T}|\boldsymbol{X}_{1:T})} = \frac{1/(3N|R^*|)}{1/(3N|A||B|)} = \frac{|A||B|}{|R^*|}.$$

The reverse step to a remove event is another add event, so the proposal ratio is

$$Q = \frac{q(\boldsymbol{X}_{1:T}|\boldsymbol{X}^*_{1:T})}{q(\boldsymbol{X}^*_{1:T}|\boldsymbol{X}_{1:T})} = \frac{1/(3N|A^*||B^*|)}{1/(3N|R|)} = \frac{|R|}{|A^*||B^*|}.$$

Then the acceptance rate is

$$\alpha(\boldsymbol{X}_{1:T}, \boldsymbol{X}^*_{1:T}) = \min\left\{1, \frac{\pi(\boldsymbol{X}^*_{1:T}|\boldsymbol{\theta}, \boldsymbol{x}_0, \boldsymbol{Y})}{\pi(\boldsymbol{X}_{1:T}|\boldsymbol{\theta}, \boldsymbol{x}_0, \boldsymbol{Y})} Q\right\},$$

which we calculate using Equation S2.

Each move in $\boldsymbol{X}_{1:T}$ will only change the latent variables of one individual (for no more than m time-steps). Hence we run the latent variable update step many times within each overall MCMC iteration (as in the Rippler algorithm). The full method is shown in Algorithm S1.

S2.2 Simulation Study

We now assess the performance of the reversible-jump algorithm by attempting to recover the parameters for the simulated data. We use the same data as when we performed parameter recovery using the Rippler algorithm in the main text.

Algorithm S1 Reversible-jump MCMC

Require: Number of iterations K, number of iterations for latent updates K', starting values $\boldsymbol{\theta}^{(0)}, \boldsymbol{X}^{(0)}, \boldsymbol{\Sigma}^{(0)}, \kappa^{(0)}$, escape probability ε, prior distribution hyperparameters $\boldsymbol{\mu}$, recovery rate γ, initial probability of colonisation p_0, test data $\boldsymbol{Y}$, maximum block update size m.

Let $\boldsymbol{\theta} = \boldsymbol{\theta}^{(0)}, \boldsymbol{X} = \boldsymbol{X}^{(0)}, \boldsymbol{\Sigma} = \boldsymbol{\Sigma}^{(0)}, \kappa = \kappa^{(0)}$.
for $k \in \{1, \dots, K\}$ **do**
  With probability $1 - \varepsilon$, sample $\boldsymbol{\theta}^* \sim \text{Normal}(\boldsymbol{\theta}, \kappa^2 \boldsymbol{\Sigma})$. Otherwise, sample $\boldsymbol{\theta}^* \sim \text{Normal}(\boldsymbol{\theta}, \boldsymbol{\Sigma}^{(0)})$.
  Calculate
$$\alpha(\boldsymbol{\theta}, \boldsymbol{\theta}^*) = \min\left\{1, \frac{\pi(\boldsymbol{\theta}^* | \boldsymbol{X}, \boldsymbol{Y})}{\pi(\boldsymbol{\theta} | \boldsymbol{X}, \boldsymbol{Y})}\right\}.$$
  Let $\boldsymbol{\theta} = \boldsymbol{\theta}^*$ with probability $\alpha(\boldsymbol{\theta}, \boldsymbol{\theta}^*)$. Otherwise keep $\boldsymbol{\theta}$ constant.
  Update κ and $\boldsymbol{\Sigma}$.
  Let $\boldsymbol{x}_0^* = \boldsymbol{x}_0$. Randomly choose $j \in \{1, \dots, N\}$ and let $x_{0,j}^* = 1 - x_{0,j}$.
  Calculate
$$\alpha(\boldsymbol{x}_0, \boldsymbol{x}_0^*) = \min\left\{1, \frac{\pi(\boldsymbol{x}_0^* | \boldsymbol{\theta}, \boldsymbol{X}_{1:T}, \boldsymbol{Y})}{\pi(\boldsymbol{x}_0 | \boldsymbol{\theta}, \boldsymbol{X}_{1:T}, \boldsymbol{Y})}\right\}.$$
  Let $\boldsymbol{x}_0 = \boldsymbol{x}_0^*$ with probability $\alpha(\boldsymbol{x}_0, \boldsymbol{x}_0^*)$. Otherwise keep $\boldsymbol{x}_0$ constant.
  for $k' \in \{1, \dots, K'\}$ **do**
    Randomly choose an individual $j \in \{1, \dots, N\}$.
    Randomly choose between the following options: move, add, remove.
    Follow the respective method on individual j to generate $\boldsymbol{X}_{1:T}^*$ and Q.
    Calculate
$$\alpha(\boldsymbol{X}_{1:T}, \boldsymbol{X}_{1:T}^*) = \min\left\{1, \frac{\pi(\boldsymbol{X}_{1:T}^* | \boldsymbol{\theta}, \boldsymbol{x}_0, \boldsymbol{Y})}{\pi(\boldsymbol{X}_{1:T} | \boldsymbol{\theta}, \boldsymbol{x}_0, \boldsymbol{Y})} Q\right\}.$$
    Let $\boldsymbol{X}_{1:T} = \boldsymbol{X}_{1:T}^*$ with probability $\alpha(\boldsymbol{X}_{1:T}, \boldsymbol{X}_{1:T}^*)$. Otherwise keep $\boldsymbol{X}$ constant.
  end for
end for

We run the MCMC algorithm for $K = 100{,}000$ iterations, repeating the latent variable step 400 times within each iteration. The first 10,000 iterations are discarded as burn-in. We let $\boldsymbol{\theta}^{(0)} = (0.5, 0.5, 0, 0)$, and then generate $\boldsymbol{X}^{(0)}$ from our transmission model using parameters $\boldsymbol{\theta}^{(0)}$. The rate of the prior distribution for each parameter is set to 0.001, such that the prior is uninformative. These are all the same values as when we ran the Rippler algorithm in the main text (to make the comparison as fair as possible). The maximum block update size is set to $m = 4$; we are assuming a recovery rate of $\gamma = 0.5$, so more than 85% of colonisation periods will be less than or equal to 4 weeks.

The parameter trace plots and histograms are shown in Figures S10 and S11 respectively. We see that the true value of each parameter lies within the 95% credible interval of the sample. Details are given in Table S2. Similarly to the results with the Rippler algorithm, the median of the sample is very close to the true value for β_G and β_H, but significantly further away for δ_A and δ_S. The central 95% credible interval for the number of individuals colonised at each point in time is shown in Figure S12, with the true value mostly lying within the interval. We can see from the trace plots of the parameters that the mixing is worse than when using the Rippler algorithm. The higher autocorrelation is because of the slow movement in the latent variable

space when using the reversible-jump algorithm. A potential fix for this would be increasing the number of reversible-jump updates per MCMC iteration, although this has the cost of increasing the runtime.

Table S2: Parameter estimates for simulated 3GC-R *E. coli* test data using the reversible-jump algorithm for inference.

Parameter	Definition	True	Median	95% CrI
β_G	global transmission rate (week^{-1})	0.1	0.0791	(0.0535, 0.111)
β_H	relative within-household transmission	1.5	2.12	(1.39, 3.51)
δ_A	age covariate effect (year^{-1})	0	-0.00410	(-0.0130, 0.00470)
δ_S	sex covariate effect	0	0.188	(-0.118, 0.501)

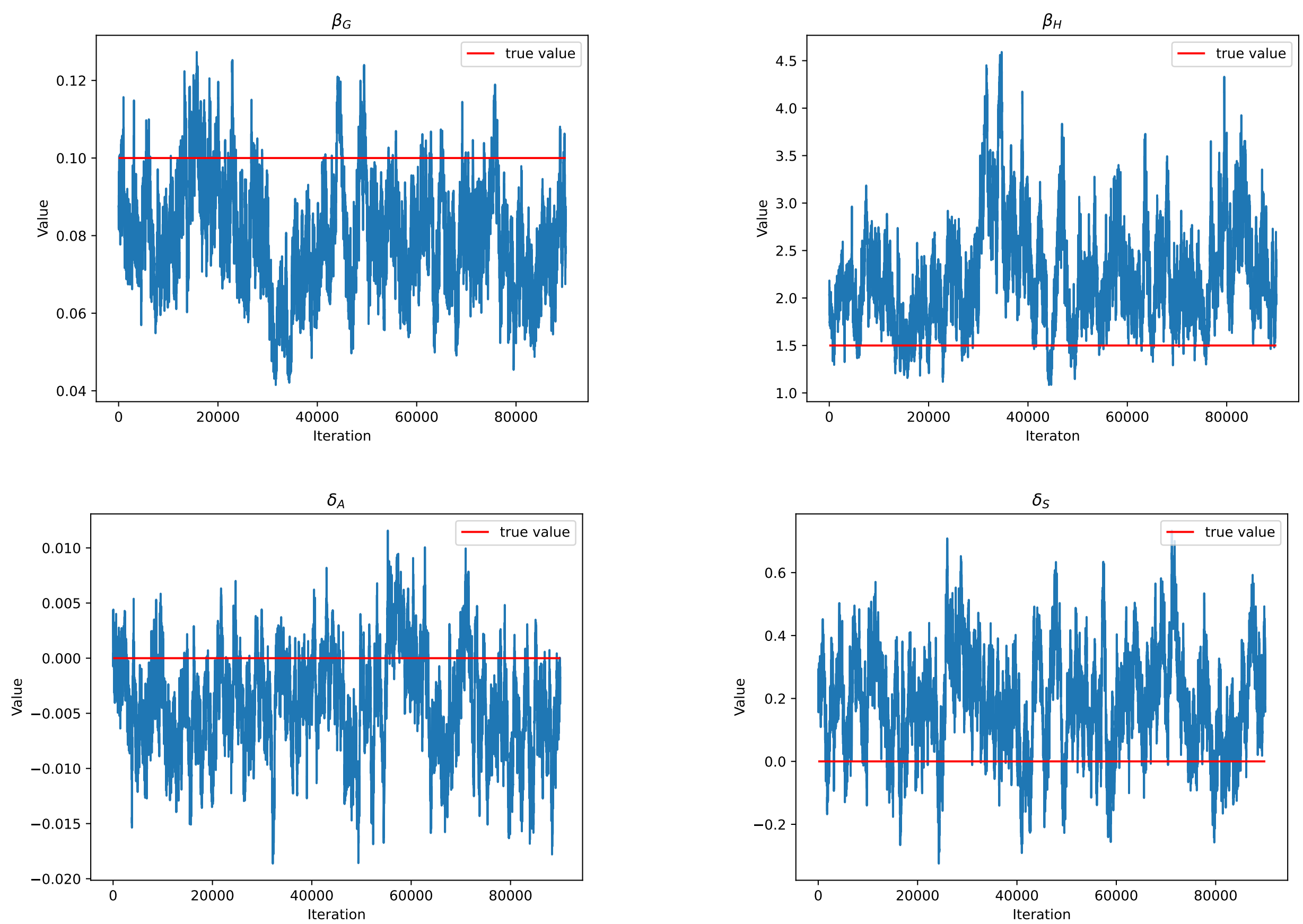

Figure S10: Trace plots of the posterior samples generated for the parameters $\boldsymbol{\theta} = (\beta_G, \beta_H, \delta_A, \delta_S)$ for simulated 3GC-R *E. coli* test data (using the reversible-jump algorithm for inference). The true value is shown by the red line.

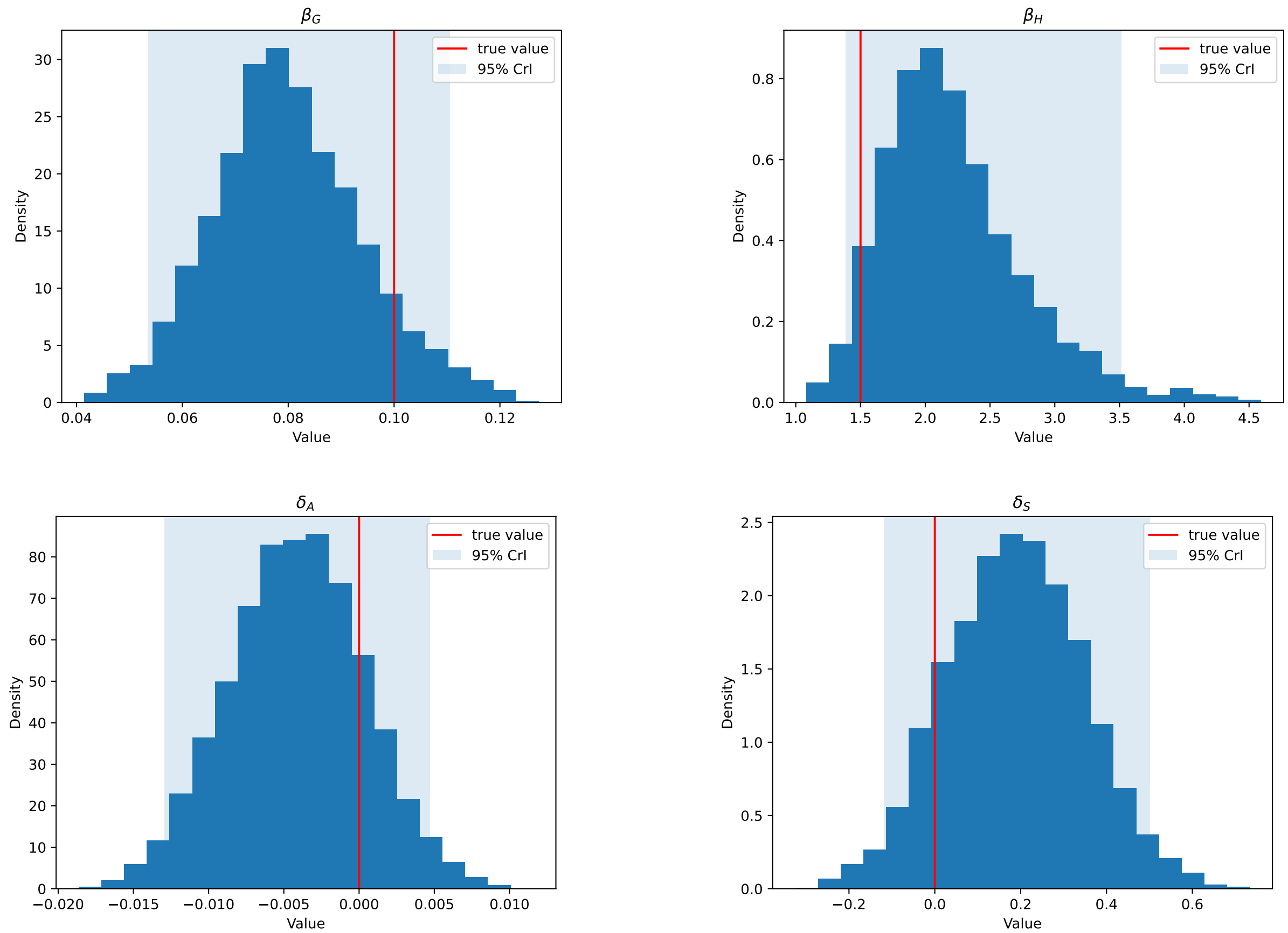

Figure S11: Histograms of the posterior samples generated for the parameters $\boldsymbol{\theta} = (\beta_G, \beta_H, \delta_A, \delta_S)$ for simulated 3GC-R *E. coli* test data using the reversible-jump algorithm (Algorithm S1). The true value is shown by the red line and the 95% credible interval is shown in light blue.

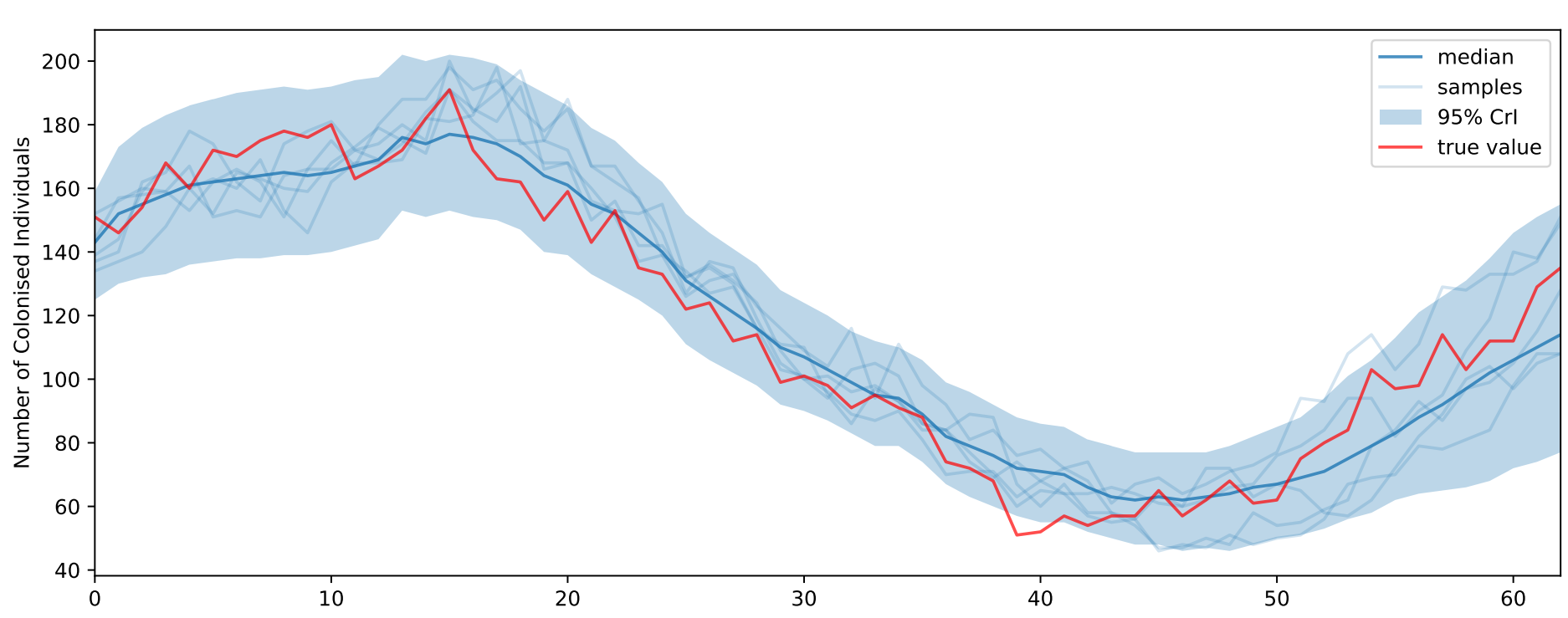

Figure S12: The number of colonised individuals over time for simulated 3GC-R *E. coli* test data inferred using the reversible-jump algorithm. The median value is shown by the dark blue line and 5 randomly chosen samples are shown by the faint blue lines. The true value is shown by the red line and the 95% credible interval is shown in light blue.

S3 iFFBS

S3.1 Inference Algorithm

We use the method from Touloupou et al. (2020) to perform individual forward filtering backwards sampling (iFFBS). As with the Rippler algorithm, we update our parameters and latent variables separately: a Metropolis-within-Gibbs step for the parameters, and a Gibbs step for the latent variables. We use the same method of updating the parameters $\boldsymbol{\theta}$ as in the Rippler algorithm (adaptive random walk Metropolis).

iFFBS is an extension of the standard FFBS algorithm: instead of sampling all of the latent variables, the latent variables for one individual are sampled conditionally on the latent variables for all other individuals (which are held constant). We randomly choose an individual j to have their latent variables updated, and let $\boldsymbol{X}_{0:T,-j}$ consist of the latent variables for all individuals other than j. We sample directly from the conditional distribution $\pi(\boldsymbol{X}_{0:T,j}|\boldsymbol{\theta}, \boldsymbol{X}_{0:T,-j}, \boldsymbol{Y})$, so we do not need to perform a Metropolis-Hastings accept-reject step.

From Bayes' theorem we have

$$\begin{aligned}
\pi(\boldsymbol{X}_{0:T,j}|\boldsymbol{\theta}, \boldsymbol{X}_{0:T,-j}, \boldsymbol{Y}) &= \pi(X_{T,j}|\boldsymbol{\theta}, \boldsymbol{X}_{0:T,-j}, \boldsymbol{Y}_{0:T,j}) \\
&\quad \prod_{t=0}^{T-1} \pi(X_{t,j}|\boldsymbol{\theta}, X_{t+1,j}, \boldsymbol{X}_{0:t+1,-j}, \boldsymbol{Y}_{0:t,j}) \\
&\propto \pi(X_{T,j}|\boldsymbol{\theta}, \boldsymbol{X}_{0:T,-j}, \boldsymbol{Y}_{0:T,j}) \\
&\quad \prod_{t=0}^{T-1} \pi(X_{t+1,j}|\boldsymbol{\theta}, \boldsymbol{X}_t) \pi(X_{t,j}|\boldsymbol{\theta}, \boldsymbol{X}_{0:t+1,-j}, \boldsymbol{Y}_{0:t,j}),
\end{aligned}$$

using the fact that the state of any individual at time $t+1$ is determined based on the state of all individuals at time t. We will iteratively calculate the probabilities $\pi(X_{t,j}|\boldsymbol{\theta}, \boldsymbol{X}_{0:t+1,-j}, \boldsymbol{Y}_{0:t,j})$ forwards through time, and then combine this term with $\pi(X_{t+1,j}|\boldsymbol{\theta}, \boldsymbol{X}_t)$ to sample backwards through time. Note that from our transmission model, equation 2 in the main text, we have

$$\mathbb{P}(X_{t+1,j} = x_{t+1,j}|\boldsymbol{\theta}, X_{t,j} = x_{t,j}, \boldsymbol{X}_{t,-j}) = \begin{cases} e^{-\lambda_j(t+1,\boldsymbol{x}_t,\boldsymbol{\theta})} & \text{if } x_{t,j} = 0, x_{t+1,j} = 0, \\ 1 - e^{-\lambda_j(t+1,\boldsymbol{x}_t,\boldsymbol{\theta})} & \text{if } x_{t,j} = 0, x_{t+1,j} = 1, \\ 1 - e^{-\gamma} & \text{if } x_{t,j} = 1, x_{t+1,j} = 0, \\ e^{-\gamma} & \text{if } x_{t,j} = 1, x_{t+1,j} = 1, \end{cases} \tag{S3}$$

for any $t \in \{0, \ldots, T-1\}$ and $j \in \{1, \ldots, N\}$.

First, we calculate the forward probabilities for $t = 0$. We have

$$\mathbb{P}(X_{0,j} = x_{0,j}|\boldsymbol{\theta}, \boldsymbol{X}_{0:1,-j}) \propto \mathbb{P}(X_{0,j} = x_{0,j}) \prod_{i=1, i\neq j}^{N} \pi(X_{1,i} = x_{1,i}|\boldsymbol{\theta}, X_{0,i} = x_{0,i}, \boldsymbol{X}_{0,-i}). \tag{S4}$$

The first term is the probability of the initial conditions, which is

$$\mathbb{P}(X_{0,j} = x_{0,j}) = \begin{cases} p_0 & \text{if } x_{0,j} = 1, \\ 1 - p_0 & \text{if } x_{0,j} = 0, \end{cases}$$

and the second term is the transition probabilities for all individuals other than j at time $t = 1$, which we can calculate using Equation S3. We calculate these probabilities up to some constant of proportionality using these equations for $x_{0,j} \in \{0, 1\}$, and then normalise such that the probabilities add to 1.

Next, we calculate the forward probabilities iteratively for $t \in \{1, \ldots, T-1\}$. We have

$$\begin{aligned}\mathbb{P}(X_{t,j} = x_{t,j}|\boldsymbol{\theta}, \boldsymbol{X}_{0:t+1,-j}, \boldsymbol{Y}_{0:t,j}) \propto\ & \mathbb{P}(Y_{t,j} = y_{t,j}|X_{t,j} = x_{t,j}) \\ & \prod_{i=1, i\neq j}^{N} \pi(X_{t+1,i} = x_{t+1,i}|\boldsymbol{\theta}, X_{t,i} = x_{t,i}, \boldsymbol{X}_{t,-i}) \\ & \mathbb{P}(X_{t,j} = x_{t,j}|\boldsymbol{\theta}, \boldsymbol{X}_{1:t,-j}, \boldsymbol{Y}_{1:t-1,j}).\end{aligned} \tag{S5}$$

The first term is the probability of the test result given the true state of the individual, which is

$$\mathbb{P}(Y_{t,j} = y_{t,j}|X_{t,j} = x_{t,j}) = \begin{cases} s_e & \text{if } y_{t,j} = 1, x_{t,j} = 1, \\ 1 - s_p & \text{if } y_{t,j} = 1, x_{t,j} = 0, \\ 1 - s_e & \text{if } y_{t,j} = 0, x_{t,j} = 1, \\ s_p & \text{if } y_{t,j} = 0, x_{t,j} = 0, \\ 1 & \text{if } y_{t,j} \text{ is undefined.} \end{cases}$$

Note that we let this term equal 1 if there is no test result at this time, so the term has no effect on the calculation of the probabilities. The second term is the transition probabilities for all individuals other than j at time $t+1$, which we can calculate using Equation S3. By the law of total probability, the third term can be expanded into

$$\begin{aligned}&\mathbb{P}(X_{t,j} = x_{t,j}|\boldsymbol{\theta}, \boldsymbol{X}_{1:t,-j}, \boldsymbol{Y}_{1:t-1,j}) \\ &= \mathbb{P}(X_{t_j} = x_{t,j}|\boldsymbol{\theta}, X_{t-1,j} = 0, \mathbf{X}_{t-1,-j})\mathbb{P}(X_{t-1,j} = 0|\boldsymbol{\theta}, \boldsymbol{X}_{0:t,-j}, \boldsymbol{Y}_{0:t-1,j}) \\ &\quad + \mathbb{P}(X_{t_j} = x_{t,j}|\boldsymbol{\theta}, X_{t-1,j} = 1, \mathbf{X}_{t-1,-j})\mathbb{P}(X_{t-1,j} = 1|\boldsymbol{\theta}, \boldsymbol{X}_{0:t,-j}, \boldsymbol{Y}_{0:t-1,j}),\end{aligned}$$

which we can calculate using Equation S3 and using the forward probabilities from the previous time-step. Again, we calculate these probabilities up to some constant of proportionality using these equations for $x_{t,j} \in \{0, 1\}$, and then normalise such that the probabilities add to 1.

The final forward probability calculation we need to determine is for $t = T$. We have

$$\begin{aligned}\mathbb{P}(X_{T,j} = x_{T,j}|\boldsymbol{\theta}, \boldsymbol{X}_{0:T,-j}, \boldsymbol{Y}_{0:T,j}) \propto\ & \mathbb{P}(Y_{T,j} = y_{T,j}|X_{T,j} = x_{T,j}) \\ & \mathbb{P}(X_{T,j} = x_{T,j}|\boldsymbol{\theta}, \boldsymbol{X}_{1:T,-j}, \boldsymbol{Y}_{1:T-1,j}).\end{aligned} \tag{S6}$$

Calculating the forward probabilities is the same as for $t \in \{1, \ldots, T-1\}$, but with one term removed.

Now we can simulate new values for $\boldsymbol{X}_{0:T,j}$ backwards through time. First, we let

$$p = \mathbb{P}(X_{T,j} = 1|\boldsymbol{\theta}, \boldsymbol{X}_{0:T,-j}, \boldsymbol{Y}_{0:T,j}),$$

and then sample $x^*_{T,j} \sim \text{Bernoulli}(p)$.

Then for $t \in \{T-1, \ldots, 0\}$, we calculate

$$\begin{aligned}\mathbb{P}(X_{t,j} = x_{t,j}|\boldsymbol{\theta}, X_{t+1,j} = x^*_{t+1,j}, \boldsymbol{X}_{0:t+1,-j}, \boldsymbol{Y}_{0:t,j}) \propto\ & \mathbb{P}(X_{t+1,j} = x^*_{t+1,j}|\boldsymbol{\theta}, X_{t,j} = x_{t,j}, \boldsymbol{X}_{t,-j}) \\ & \mathbb{P}(X_{t,j} = x_{t,j}|\boldsymbol{\theta}, \boldsymbol{X}_{0:t+1,-j}, \boldsymbol{Y}_{0:t,j}).\end{aligned} \tag{S7}$$

The first term is calculated using Equation S3, the second term is the forward probability for time t – we calculate for $x_{t,j} \in \{0, 1\}$ and then normalise such that the probabilities add to 1. Next, we let

$$p = \mathbb{P}(X_{t,j} = 1|\boldsymbol{\theta}, X_{t+1,j} = x^*_{t+1,j}, \boldsymbol{X}_{0:t+1,-j}, \boldsymbol{Y}_{0:t,j}),$$

and then sample $x^*_{t,j} \sim \text{Bernoulli}(p)$.

We now have new latent variables $\boldsymbol{X}_{0:T,j} = \boldsymbol{x}^*_{0:T,j}$. Each iFFBS update will only change the latent variables of one individual, so we will run the latent variable update step many times within each overall MCMC iteration (as in the Rippler algorithm). Note than in Touloupou et al. (2020), one iteration of the iFFBS algorithm performs a latent variable update on every individual (in a pre-defined order from 1 to N). We instead choose individuals to update randomly, with an arbitrary number of latent variable updates per overall MCMC iteration. This ensures a fair comparison with the RJMCMC and Rippler algorithms. The full method is shown in Algorithm S2.

Algorithm S2 iFFBS

Require: Number of iterations K, number of iterations for latent updates K',starting values $\boldsymbol{\theta}^{(0)}, \boldsymbol{X}^{(0)}, \boldsymbol{\Sigma}^{(0)}, \kappa^{(0)}$, escape probability ε, prior distribution hyperparameters $\boldsymbol{\mu}$, tuning parameter λ, recovery rate γ, initial probability of colonisation p_0, covariate data $\boldsymbol{c}$, test data $\boldsymbol{Y}$.

Let $\boldsymbol{\theta} = \boldsymbol{\theta}^{(0)}, \boldsymbol{X} = \boldsymbol{X}^{(0)}, \boldsymbol{\Sigma} = \boldsymbol{\Sigma}^{(0)}, \kappa = \kappa^{(0)}$.
for $k \in \{1, \ldots, K\}$ **do**
    With probability $1 - \varepsilon$, sample $\boldsymbol{\theta}^* \sim \text{Normal}(\boldsymbol{\theta}, \kappa^2\boldsymbol{\Sigma})$. Otherwise, sample $\boldsymbol{\theta}^* \sim \text{Normal}(\boldsymbol{\theta}, \boldsymbol{\Sigma}^{(0)})$.
    Calculate

$$\alpha(\boldsymbol{\theta}, \boldsymbol{\theta}^*) = \min\left\{1, \frac{\pi(\boldsymbol{\theta}^*|\boldsymbol{X}, \boldsymbol{Y})}{\pi(\boldsymbol{\theta}|\boldsymbol{X}, \boldsymbol{Y})}\right\}.$$

    Let $\boldsymbol{\theta} = \boldsymbol{\theta}^*$ with probability $\alpha(\boldsymbol{\theta}, \boldsymbol{\theta}^*)$. Otherwise keep $\boldsymbol{\theta}$ constant.
    Update κ and $\boldsymbol{\Sigma}$.
    for $k' \in \{1, \ldots, K'\}$ **do**
        Randomly choose an individual $j \in \{1, \ldots, N\}$.
        Calculate forward probabilities for $x_{0,j} \in \{0, 1\}$ using Equation S4.
        for $t \in \{1, \ldots, T - 1\}$ **do**
            Calculate forward probabilities for $x_{t,j} \in \{0, 1\}$ using Equation S5.
        end for
        Calculate forward probabilities for $x_{T,j} \in \{0, 1\}$ using Equation S6.
        Let $p = \mathbb{P}(X_{T,j} = 1|\boldsymbol{\theta}, \boldsymbol{X}_{0:T,-j}, \boldsymbol{Y}_{0:T,j})$.
        Sample $x^*_{T,j} \sim \text{Bernoulli}(p)$.
        for $t \in \{T - 1, \ldots, 0\}$ **do**
            Calculate backwards probabilities for $x_{t,j} \in \{0, 1\}$ using Equation S7.
            Let $p = \mathbb{P}(X_{t,j} = 1|\boldsymbol{\theta}, X_{t+1,j} = x^*_{t+1,j}, \boldsymbol{X}_{0:t+1,-j}, \boldsymbol{Y}_{0:t,j})$.
            Sample $x^*_{t,j} \sim \text{Bernoulli}(p)$.
        end for
        Let $\boldsymbol{X}_{0:T,j} = \boldsymbol{x}^*_{0:T,j}$.
    end for
end for

S3.2 Simulation Study

We now assess the performance of the iFFBS algorithm by attempting to recover the parameters for the simulated data. We use the same data as when we performed parameter recovery using the Rippler algorithm in the main text.

We run the MCMC algorithm for $K = 100{,}000$ iterations, repeating the latent variable (iFFBS) step 400 times within each iteration. The first 10,000 iterations are discarded as burn-in. We let $\boldsymbol{\theta}^{(0)} = (0.5, 0.5, 0, 0)$, and then generate $\boldsymbol{X}^{(0)}$ from our transmission model using parameters $\boldsymbol{\theta}^{(0)}$. The rate of the prior distribution for each parameter is set to 0.001. For consistency we use all the same values as when we ran the Rippler algorithm in the main text.

The parameter trace plots and histograms are shown in Figures S13 and S14 respectively. Details are given in Table S3. Clearly from the trace plots we see that the mixing is better than when using the reversible-jump algorithm. We see that the true value of each parameter lies within the 95% credible interval of the sample. Similarly to the results with the Rippler algorithm, the median of the sample is very close to the true value for β_G and β_H, but significantly further away for δ_A and δ_S. The central 95% credible interval for the number of individuals colonised at each point in time is shown in Figure S15, with the true value mostly lying within the interval. We can see from the trace plots of the parameters that the mixing is at least as good as when using the Rippler algorithm.

Table S3: Parameter estimates for simulated 3GC-R *E. coli* test data using the iFFBS algorithm for inference.

Parameter	Definition	True	Median	95% CrI
β_G	global transmission rate (week^{-1})	0.1	0.0945	(0.0673, 0.132)
β_H	relative within-household transmission	1.5	1.75	(1.09, 2.75)
δ_A	age covariate effect (year^{-1})	0	-0.00415	(-0.0132, 0.00397)
δ_S	sex covariate effect	0	0.212	(-0.110, 0.513)

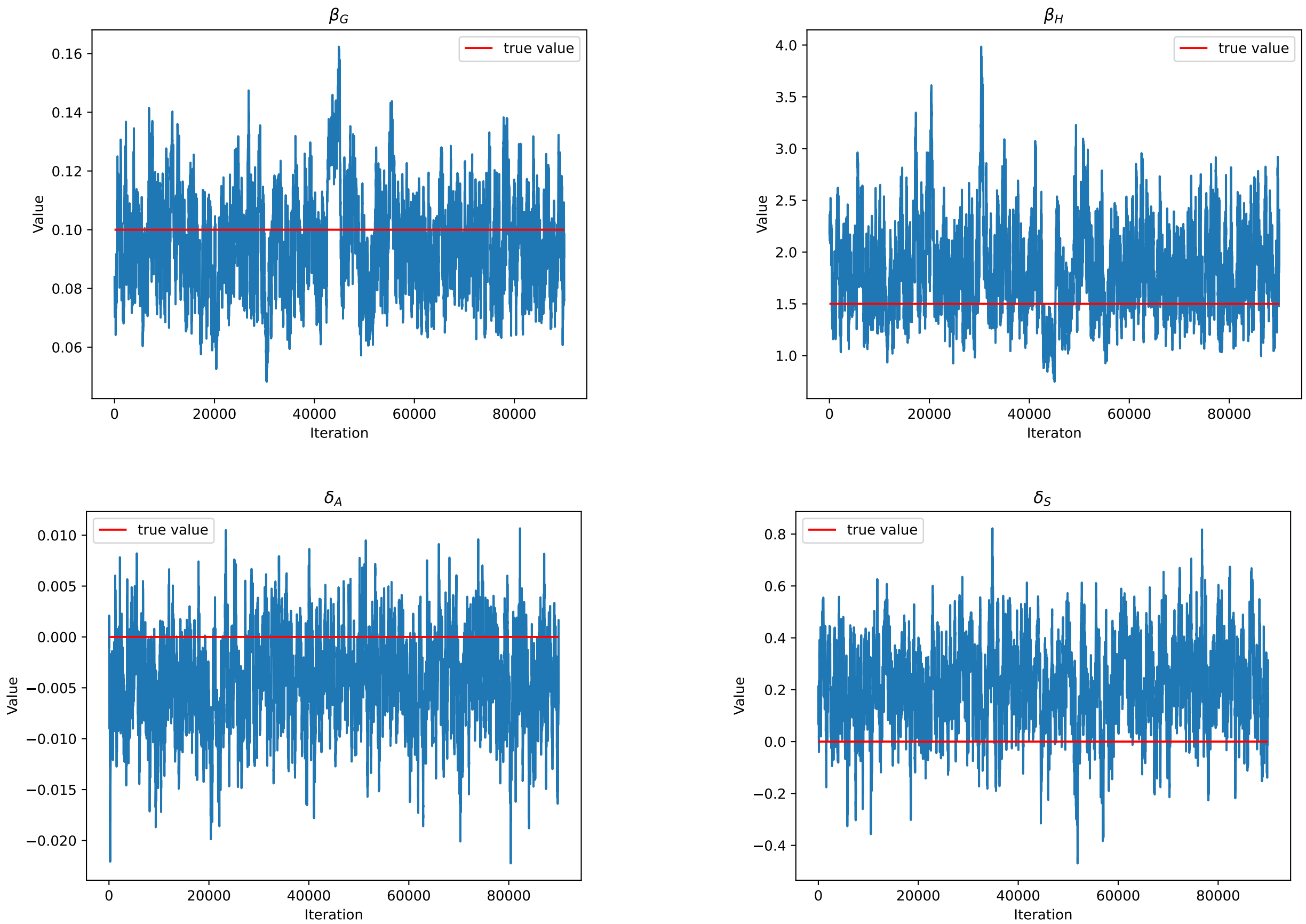

Figure S13: Trace plots of the posterior samples generated for the parameters $\boldsymbol{\theta} = (\beta_G, \beta_H, \delta_A, \delta_S)$ for simulated 3GC-R *E. coli* test data using the iFFBS algorithm (Algorithm S2). The true value is shown by the red line.

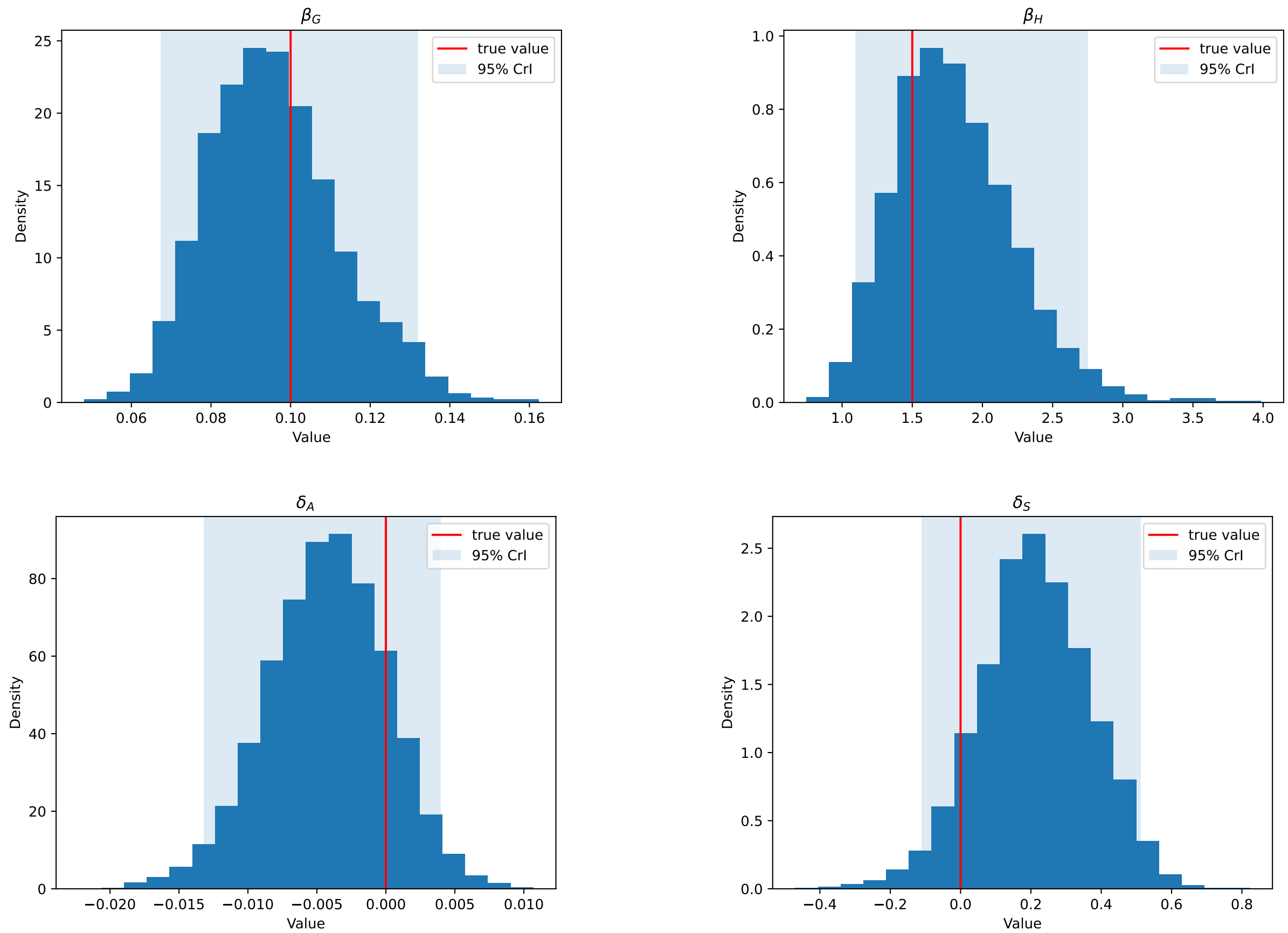

Figure S14: Histograms of the posterior samples generated for the parameters $\boldsymbol{\theta} = (\beta_G, \beta_H, \delta_A, \delta_S)$ for simulated 3GC-R *E. coli* test data using the iFFBS algorithm. The true value is shown by the red line and the 95% credible interval is shown in light blue.

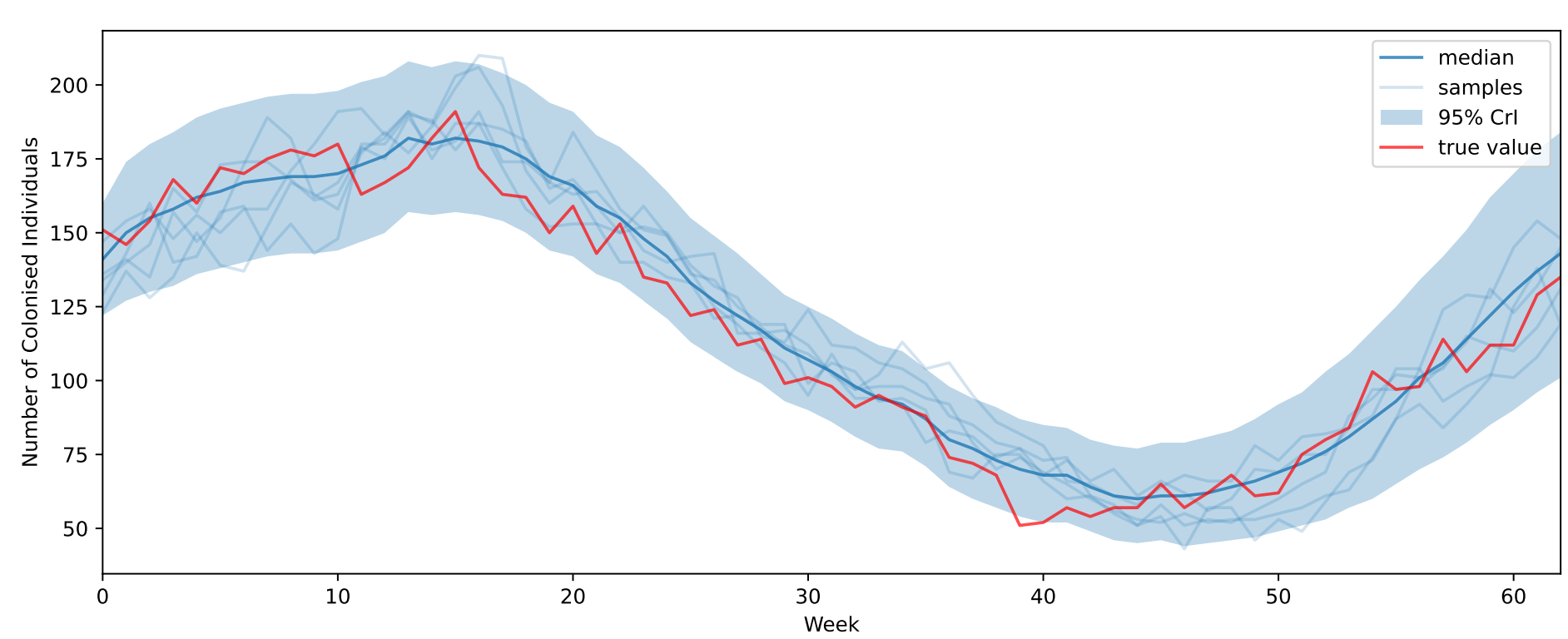

Figure S15: The number of colonised individuals over time for simulated 3GC-R *E. coli* test data inferred using the iFFBS algorithm. The median value is shown by the dark blue line and 5 randomly chosen samples are shown by the faint blue lines. The true value is shown by the red line and the 95% credible interval is shown in light blue.
